# Diagnostic value of monocyte chemoattractant Protein-1, soluble mannose receptor, Presepsin, and Procalcitonin in critically ill children admitted with suspected sepsis

**DOI:** 10.1186/s12887-021-02930-7

**Published:** 2021-10-19

**Authors:** Noha A. Hassuna, Ebtesam Elgezawy, Suzan O. Mousa, Reem A. AbdelAziz, Reham A. Ibrahem, Wafaa Yousif Abdel Wahed, Khalid A. Nasif, Enas M. Hefzy

**Affiliations:** 1 Medical Microbiology and Immunology Department, Faculty of Medicine, Minia University, Minia, Egypt; 2 Clinical Pathology Department, Faculty of Medicine, Assiut University, Asyut, Egypt; 3 Pediatrics Department, Faculty of Medicine, Minia University, Minia, 61111 Egypt; 4 Microbiology and Immunology, Faculty of Pharmacy, Minia University, Minia, Egypt; 5 Public Health Department, Faculty of Medicine, Fayoum University, Faiyum, Egypt; 6 Biochemistry Department, Faculty of Medicine, Minia University, Minia, Egypt; 7 Clinical Biochemistry, Faculty of Medicine, King Khaled University, Abha, Saudi Arabia; 8 Medical Microbiology and Immunology Department Faculty of Medicine, Fayoum University, Faiyum, Egypt

**Keywords:** Sepsis, Presepsin, Procalcitonin, SIRS, Soluble mannose receptor

## Abstract

**Introduction:**

The differentiation between systemic inflammatory response syndrome and sepsis is very important as it determines essential treatment decisions, such as selection, initiation, and duration of antibiotic therapy.

**Objectives:**

We aimed to investigate the diagnostic value of Procalcitonin, Monocyte Chemoattractant Protein-1, soluble Mannose Receptor, Presepsin as early biomarkers of pediatric sepsis in comparison to systemic inflammatory response syndrome in severely ill children.

**Patients and methods:**

This study included 58 children diagnosed as sepsis (group 1), 24 children with systemic inflammatory response syndrome without infection (group 2), and 50 healthy children as controls (group 3). All the plasma levels of the studied biomarkers were measured and ROC curves were created for all the tested parameters to discriminate between sepsis and SIRS.

**Results:**

The area under the curve for Monocyte Chemoattractant Protein-1 was 0.926 (0.846-0.927) with sensitivity 100% and specificity 62.5%. The soluble Mannose Receptor had the highest sensitivity (100%), with AUC equals 1(.0.956-1.0) and specificity of 100%. The cut-off values for Procalcitonin, Presepsin, soluble Mannose Receptor, and Monocyte Chemoattractant Protein-1 and were: 0.62 ng/ml, 100 pg/ml, 13 ng/ml and 90 pg/ml, respectively. In septic cases, both soluble Mannose Receptor and Procalcitonin have positive correlations with the severity of sepsis, low Glasgow Coma Scale, ventilatory support, use of inotropic drugs and mortality rate (r = 0.950, 0.812, 0.795, 0.732 and 0.861respectively) for soluble Mannose Receptor and (0.536, 0.473, 0.422, 0.305 and 0.474 respectively) for Procalcitonin.

**Conclusion:**

Soluble Mannose Receptor, Presepsin, and Monocyte Chemoattractant Protein-1 can be used to differentiate between sepsis and SIRS in critically ill children.

## What is known?

Procalcitonin, Monocyte Chemoattractant Protein-1 (MCP-1), soluble Mannose Receptor (sMR) and Presepsin are known to be useful biomarkers in the diagnosis of sepsis.

## What is new?

- We used these markers to investigate their diagnostic significance in severely ill children admitted with signs of acute and severe inflammation and correlate these markers to the severity of sepsis.
- We also assess whether these biomarkers are useful to discriminate sepsis from SIRS in critically ill children with suspected infection.

## Introduction

The recent Sepsis-3 consensus definition emphasized that sepsis is differentiated from uncomplicated infection by the presence of life-threatening organ dysfunction as a result of a dysregulated host response to infection [1].

Systemic inflammatory response syndrome (SIRS) is defined as an inflammatory condition involving the entire body. That is the reaction of the body to an insult that is either contagious or non-contagious such as burns, autoimmune disorders, pancreatitis, trauma, and other conditions [2].

Sepsis is difficult to be distinguished from other non-infectious situations in critically ill patients presented with clinical signs of severe inflammation. This topic has a vital importance as the management differs greatly in these two different conditions. Thus, there is an unfulfilled requirement for clinical or laboratory diagnostic tools to distinguish between SIRS and various forms of sepsis [3].

Differentiation between SIRS and sepsis is very important as it determines essential treatment decisions, such as selection, initiation, and duration of antibiotic therapy [4].

As SIRS is diagnosed clinically while sepsis is further diagnosed by the presence or suspected infection, it is essential to develop useful biomarkers to confirm or eliminate the suspected infection, stage of sepsis, make a therapeutic decision, as well as monitor response to treatment [5].

Procalcitonin (PCT), a precursor for calcitonin, is produced by all body tissues [6]. It has been reported that PCT serum levels are elevated with sepsis in pediatric population in addition to other conditions as cancer, autoimmune disease, viral, parasitic infection, and tissue necrosis [7–9].

Monocyte Chemoattractant Protein-1 (MCP-1) is a strong chemoattractant of mononuclear cells and a regulatory mediator in sepsis. MCP-1 has an essential immunomodulatory function to maintain the balance between pro-inflammatory and anti-inflammatory cytokines in sepsis. Elevated concentrations of MCP-1 were reported in adult patients with sepsis and septic shock [10].

The soluble Mannose Receptor (sMR) is expressed primarily by subsets of macrophages, dendritic and endothelial cells. The sMR is shed from the cell surface due to inflammation, and the receptor can be detected in the blood [11]. It is a new possible sepsis biomarker in adults; however, it is not yet tested as a biomarker for sepsis in pediatrics [12].

Presepsin, (sCD14-subtypes) was found to have a value in the diagnosis of sepsis. CD14 can generate a strong and exaggerated systemic inflammatory reaction and activate the fibrinolytic and coagulation systems, leading to SIRS, disseminated intravascular coagulation (DIC), multiple organ dysfunction syndrome (MODS), and septic shock [13].

CD14 can also turn on a sequence of inflammatory and signal transduction pathways that result in the systemic inflammatory response [14].

The aim of our study is to investigate the diagnostic significance of PCT, MCP-1, sMR, and Presepsin in severely ill children admitted with signs of acute and severe inflammation and correlate these markers to the severity of sepsis. We also assess whether these biomarkers are useful to discriminate sepsis from SIRS in critically ill newly hospital admitted children with suspected infection.

## Patients and methods

### Patients

This is a hospital-based prospective case control comparative study conducted at the Pediatric Intensive Care Unit (PICU) at Minia University Children Hospital during the period from April 2019 to November 2019. Our study included eighty-two children admitted to our PICU with signs of acute and severe inflammation (group 1 and 2) and fifty apparently healthy children as controls. According to the the 2005 Consensus definition for paediatric sepsis [15], we classified children into 3 groups:

Group (1) included 58 children diagnosed as sepsis or septic shock.

Group (2) included 24 children diagnosed as SIRS without infection.

Group (3) included 50 healthy children served as controls.

### Definitions of systemic inflammatory response syndrome (SIRS), infection, sepsis, severe sepsis, and septic shock

#### Sirs

The presence of at least two of the following four criteria, one of which must be abnormal temperature or leukocyte count:

- Core temperature of > 38.5 °C or < 36 °C.
- Tachycardia, defined as a mean heart rate > 2 SD above normal for age in the absence of external stimulus, chronic drugs, or painful stimuli; or otherwise unexplained persistent elevation over a 0.5- to 4-h time period OR for children < 1 yr old: bradycardia, defined as a mean heart rate < 10th percentile for age in the absence of external vagal stimulus, β-blocker drugs, or congenital heart disease; or otherwise unexplained persistent depression over a 0.5-h time period.
- Mean respiratory rate > 2 SD above normal for age or mechanical ventilation for an acute process not related to underlying neuromuscular disease or the receipt of general anesthesia.
- Leukocyte count elevated or depressed for age (not secondary to chemotherapy-induced leukopenia) or > 10% immature neutrophils.

#### Infection

A suspected or proven (by positive culture, tissue stain, or polymerase chain reaction test) infection caused by any pathogen OR a clinical syndrome associated with a high probability of infection. Evidence of infection includes positive findings on clinical exam, imaging, or laboratory tests (e.g., white blood cells in a normally sterile body fluid, perforated viscus, chest radiograph consistent with pneumonia, petechial or purpuric rash, or purpura fulminans).

#### Sepsis

SIRS in the presence of or as a result of suspected or proven infection.

#### Severe sepsis

Sepsis plus one of the following: cardiovascular organ dysfunction OR acute respiratory distress syndrome OR two or more other organ dysfunctions.

#### Septic shock

Sepsis and cardiovascular organ dysfunction [15].

Immediately after PICU admission, all patients with suspected sepsis were included in our study. We excluded cases whose parents refused to participate, Patients who died within the first eight hours after admission, discharge within the first 24 h after admission. Children with underlying malignancies, chronic disease or immunosuppression state were also excluded from our study.

## Methods

Studied children were subjected to thorough history taking; gender, age and cause of admission stressing on the focus of infection. General examination and vital signs monitoring (temperature, heart rate, blood pressure, Glasgow Coma Scale [GCS], qSOFA) were done for all patients [16].

A standard Pediatric Risk of Mortality score (PRISM III-24) was done during the first 24 h after admission for each septic patient to determine the severity of sepsis [17].

Both primary outcome measures (patients’ mortality) and secondary outcome measures (length of stay in PICU, ventilatory support and requirement of inotropic drugs) were also recorded.

### Blood sampling for biomarkers

Within 12 h after admission, under complete aseptic conditions, three ml blood samples were drawn from all cases for Complete Blood Count (CBC), C- reactive protein (CRP), renal, liver functions and the studied biomarkers. Plasma was collected by centrifugation at 4 °C, aliquoted, and stored at − 70 °C till assay.

Cell Dyne 3500 automated cell counter was used for complete blood count of blood collected in EDTA-containing tubes. Renal and hepatic functions; including creatinine level, direct and total bilirubin, and alanine aminotransferase (ALT) were assessed in all patients by BM Hitachi 911 Chemistry Analyzer. PCT and MCP-1 were detected in serum by bioMérieux SA kit (VIDAS, France) and BioSource immunoassay kit (BioSource International Inc., USA), respectively. Measurement of plasma concentrations of Presepsin was performed by PATHFAST immunoassay analytical system (PROGEN Biotechnik GmbH, Germany; Mitsubishi Chemical Medience Corporation, Japan). The sMR was detected by enzyme linked immunosorbent assay (Glory Science, USA).

*Reference ranges: sMR* = 1-256 ng/mL, Procalcitonin = 0.15 ng/mL

Presepsin = 320 pg/ml, MCP-1 = 1-800 pg/ml.

### Processing of blood cultures

Before initiation of antibiotics and within the first 12 h after admission, two ml of blood collected by sterile venipuncture for blood cultures. Blood cultures were done by the Bact/Alert FA (bioMérieux, Marcy l’Etoile, France).

Bacteremia was identified when blood culture had microbial growth. Positive samples were then subcultured on blood agar, MacConkey agar, and chocolate agar media. Isolated organisms were differentiated by colony morphology and Gram-staining. Identification of Gram-positive and Gram-negative bacteria was performed by the conventional biochemical reactions.

Positive blood cultures confirmed the diagnosis of bacterial sepsis. Other investigations as chest X-ray, urine cultures or deep tissue swabs were performed when needed.

### Statistical analysis

The collected data were coded, tabulated, and statistically analyzed using SPSS program (Statistical Package for Social Sciences) software version 25.

Descriptive statistics were done for parametric (normally distributed) quantitative data by mean, Standard deviation (SD) and minimum and maximum of range and for non-parametric quantitative data by median and interquartile range (IQR), while for qualitative data by frequency and percentage.

Distribution of the data was done by Kolmogorov Smirnov test and Shapiro Wilk test.

Analyses were done between the three groups for parametric quantitative data using One way ANOVA test between the three groups followed by post hoc Tukey’s analysis between each two groups and for non-parametric quantitative data using Kruskal Wallis test between the three groups followed by Mann Whitney test between each two groups.

Analyses were done between the two groups for Qualitative data using Chi square test.

Correlation was done using Pearson’s correlation coefficients.

ROC curve analysis was used to determine AUC, Sensitivity, Specificity, PPV, NPV and accuracy of variables predicting sepsis.

The level of significance was taken at (*P* value ≤0.05).

### Ethical considerations

The study had the approval of the Minia College of Medicine Ethical Committee. All the actions performed were according to the Helsinki Declaration and its modifications. Before patients’ enrollment in the study, written informed approval was obtained from their parents.

## Results

One hundred thirty-two children were included in our study. Children were classified into three groups:

- Group (1): 58 children diagnosed as having sepsis or septic shock.
- Group (2): 24 children diagnosed as having SIRS without infection.
- Group (3): 50 apparently healthy children as controls.

Demographic and clinical data are presented in Table 1. There was no statistical difference between the first two groups regarding the age, sex, length of stay, ventilatory support, Glasgow Coma Scale, the need for inotropic drugs, and outcome (Table 1).

**Table 1:** Demographic and clinical data of the studied groups

		Sepsis (I)	SIRS (II)	Control (III)	***P*** value			
		***N*** = 58	***N*** = 24	***N*** = 50	Among 3 groups	I vs II	I vs III	II vs III
Age (years)(Mean ± SD)		2.4 ± 1	2.9 ± 1.2	3 ± 0.9	*0.087*	*0.113*	*0.233*	*0.768*
Sex					*0.507*	*0.350*	*0.743*	*0.246*
M		40(69%)	19(79.2%)	33(66%)				
F		18(31%)	5(20.8%)	17(34%)				
LOS (days) (Mean ± SD)		(2-9) 5.5 ± 1.9	(2-8) 4.8 ± 1.8			*0.102*		
ventilatory Support	**No**	33(56.9%) 25(43.1%)	13(54.2%) 11(45.8%)			*0.821*		
	**Yes**							
GCS	*> 8*	38(65.5%) 20(34.5%)	12(50%) 12(50%)			*0.190*		
	*< 8*							
Inotropic drug	**No**	34(58.6%) 24(41.4%)	12(50%) 12(50%)			*0.474*		
	**Yes**							
Outcome	*Survivals*	33(56.9%) 25(43.1%)	15(62.5%) 9(37.5%)			*0.603*		
	*Non-survivals*							

One way ANOVA test for parametric quantitative data (expressed as mean ± SD) between the three groups followed by post hoc Tukey’s analysis between each two groups

Chi square test for qualitative data between groups

Significant level at *P* value < 0.05

*LOS* Length of Stay, *GCS* Glasgow Coma Scale

There was no significant difference between the studied groups regarding complete blood count elements except for total white blood cells, eosinophils, basophils, hemoglobin levels and platelet counts. Serum creatinine and CRP levels were significantly higher in sepsis group than the other two groups (*p* < 0.05) (Table 2).

**Table 2:** Laboratory data among the studied groups

		Sepsis (I)	SIRS (II)	Control (III)	P value			
		N = 58	N = 24	N = 50	Among 3 groups	I vs II	I vs III	II vs III
WBC	Mean ± SD	17.5 ± 10.2	12.1 ± 2.7	8.6 ± 1.3	< 0.001*	0.004*	< 0.001*	*0.109*
RBCs	Mean ± SD	3.9 ± 0.9	4.4 ± 0.7	4.1 ± 0.6	0.022*	0.016*	*0.595*	*0.120*
HGB	Mean ± SD	10.7 ± 2.1	12.2 ± 0.9	12.1 ± 0.5	< 0.001*	< 0.001*	< 0.001*	*0.886*
PLT	Mean ± SD	167.9 ± 71.5	302.5 ± 85.5	309.4 ± 91.5	< 0.001*	< 0.001*	< 0.001*	*0.939*
NE	Mean ± SD	66.5 ± 12.7	60.9 ± 8.7	59.8 ± 6.9	0.002*	*0.064*	0.002*	*0.895*
LY	Mean ± SD	26.7 ± 12.1	31.7 ± 8.3	32.7 ± 6.3	0.004*	*0.087*	0.005*	*0.908*
MO	Median (IQR)	6(5-8)	6(5-7)	5.3(4.5-6)	0.007*	*0.235*	0.005*	0.027*
EO	Median (IQR)	0(0-1)	2(0-2)	1.3(0.6-2.3)	< 0.001*	0.003*	< 0.001*	*0.790*
BA	Median (IQR)	0(0-0)	0(0-1)	0.5(0.3-0.7)	< 0.001*	< 0.001*	< 0.001*	*0.246*
CRP	Median (IQR)	26(22-38)	1(0.8-1.9)	1.7(1.4-2.4)	< 0.001*	< 0.001*	< 0.001*	0.021*
Serum creatinine	Mean ± SD	1.4 ± 0.7	0.9 ± 0.3	0.9 ± 0.2	< 0.001*	< 0.001*	< 0.001*	*0.987*
ALT	Median (IQR)	33(22-49)	33(19-37)	27.2(17-45)	*0.099*	*0.153*	*0.051*	*0.452*
Total bilirubin	Mean ± SD	1.7 ± 1.8	1.1 ± 0.3	1 ± 0.3	0.018*	*0.134*	0.022*	*0.977*

One way ANOVA test for parametric quantitative data (expressed as mean ± SD) between the three groups followed by post hoc Tukey’s analysis between each two groups

Kruskal Wallis test for non-parametric quantitative data (expressed as median(IQR)) between the three groups followed by Mann Whitney test between each two groups

*: Significant level at *P* value < 0.05

Sepsis secondary to pneumonia was detected in 21 cases (36.2%). 25 children (43.1%) presented with gastroenteritis. Central Nervous System (CNS) infection was detected in 12 cases (20.7%), while cases with SIRS were secondary to either trauma or hemorrhage.

Positive blood cultures were obtained in all sepsis cases. The following bacteria were isolated from the blood cultures: 16 cases (27.58%) had *Klebsiella pneumoniae,* 14 cases (24.1%) had *Staphylococcus aureus*: 12 cases (20.68%) had *Escherichia coli,* 10 cases (17.24%) had Coagulase negative staphylococcus (CONS) and 6 cases (10.3%) were *Candida albicans.* Blood cultures were negative for all SIRS cases.

### Plasma levels of PCT, sMR, MCP-1, and Presepsin

**Fig. 1:**
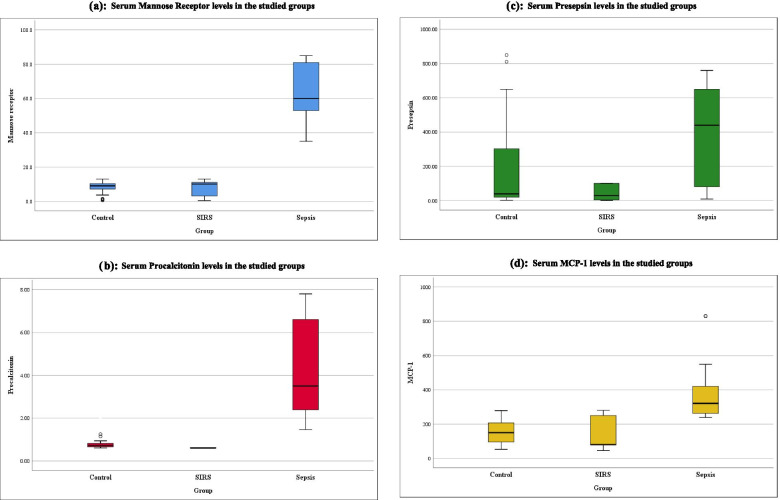
**a**: Serum Mannose Receptor levels in the studied groups. **b**: Serum Procalcitonin levels in the studied groups. **c**: Serum Presepsin levels in the studied groups. **d**: Serum MCP-1 levels in the studied groups

All plasma levels of the studied sepsis biomarkers; PCT, sMR, MCP-1, and Presepsin, increased significantly in cases with sepsis compared to both SIRS and control groups (*p <* 0.001) (Fig. 1, Table 3).

**Table 3:** Studied markers among the studied groups

		Sepsis (I)	SIRS (II)	Control (III)	P value			
		N = 58	N = 24	N = 50	Among 3 groups	I vs II	I vs III	II vs III
s-Mannose- receptor	Median (IQR)	60(53-81)	10(3.2-11)	9(7.1-10.3)	***< 0.001***	***< 0.001***	***< 0.001***	*0.308*
Procalcitonin	Median (IQR)	3.5(2.39-6.6)	0.6(0.6-0.62)	0.62(0.54-0.73)	***< 0.001***	***< 0.001***	***< 0.001***	*0.563*
Presepsin	Median (IQR)	440(80-650)	30(5-100)	40(20-313)	***< 0.001***	***< 0.001***	***< 0.001***	*0.292*
MCP-1	Median (IQR)	321 (263-420)	80 (79-250)	150 (94-207.2)	***< 0.001***	***< 0.001***	***< 0.001***	*0.525*

Kruskal Wallis test for non-parametric quantitative data (expressed as median(IQR)) between the three groups followed by Mann Whitney test between each two groups

*: Significant level at *P* value < 0.05

*Reference Ranges: sMR* = 1-256 ng/mL, Procalcitonin = 0.15 ng/mL

Presepsin = 320 pg/ml, MCP-1 = 1-800 pg/ml

### Receiver operating characteristic curve (ROC curve) analysis for prediction of sepsis

**Fig. 2:**
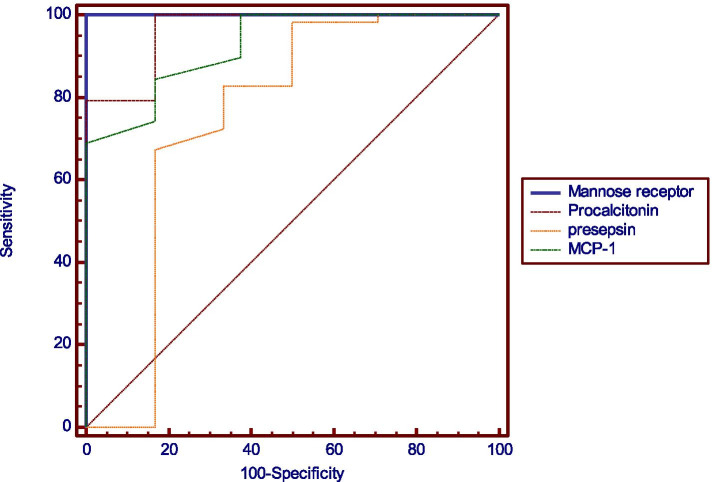
ROC curve for prediction of sepsis

ROC curves were created for all the tested parameters to discriminate between sepsis and SIRS. Area under the ROC curve (AUC) of MCP-1 was 0.926 (0.846-0.972) with sensitivity 100% and specificity 62.5% The AUC for PCT was 0.966 (0.9-0.993) with 100% sensitivity and 83.33% specificity. As regards Pesepsin; the AUC was 0.751 (0.643-0.840) with a sensitivity of 67.24% %, a specificity of 83.33%. The sMR had the highest sensitivity (100%), with AUC equals 1(0.956-1) and specificity of 100% (Fig. 2 Table 4). Both the positive and negative predictive values for the tested sepsis biomarkers are shown in (Table 4). These values were estimated for the cutoff points that have the best differentiation as obtained from the AUCs. The cut-off values for PCT, Presepsin, sMR, and MCP-1 and were: 0.62 ng/ml, 100 pg/ml, 13 ng/ml and 90 pg/ml, respectively (Table 4).

**Table 4:** ROC curve analysis for prediction of sepsis

	Mannose receptor	Procalcitonin	Presepsin	MCP-1
**AUC**	***1***	***0.966***	***0.751***	***0.926***
**95% CI**	***0.956-1***	***0.9-0.993***	***0.643-0.840***	***0.846-0.972***
**P value**	< 0.001*	< 0.001*	0.001*	< 0.001*
**Optimal cutoff point**	> 13	> 0.62	> 100	> 90
**Sensitivity**	100	100	67.24	100
**Specificity**	100	83.33	83.33	62.5
**PPV**	100	93.5	90.7	86.6
**NPV**	100	100	51.3	100
**Accuracy**	100	95.12	72	89

*AUC* Area Under the Curve, *CI* Confidence Interval, *PPV* Positive Predictive Value, *NPV* Negative Predictive Value

According to the correlation matrix between sepsis marker, positive correlation was demonstrated between PCT and MCP-1 (r = 0.663. *p* value < 0.001) and between MCP-1 and sMR (r = 0.601, *p* = 0.002) (Table 5).

**Table 5:** Correlation coefficient between the set of serum markers

SIRS group	Mannose receptor		Procalcitonin		Presepsin		MCP-1	
	r	P value	r	P value	r	P value	r	P value
**Procalcitonin**	0.174	0.417						
**Presepsin**	0.324	0.123	−0.110	0.609				
**MCP-1**	**0.601**	0.002*	**0.663**	< 0.001*	−0.291	0.168		
**CRP**	0.123	0.568	**0.796**	< 0.001*	0.296	0.161	**0.406**	0.049*

Pearson’s correlation

*: Significant level at *P* value < 0.5

In children with sepsis, both sMR and PCT have positive correlations with the severity of sepsis (PRISM III), low GCS, ventilatory support, use of inotropic drugs and mortality rate (r = 0.950, 0.812, 0.795, 0.732 and 0.861respectively) for sMR and (0.536, 0.473, 0.422, 0.305 and 0.474 respectively) for PCT. While MCP-1 was only positively correlated with PRISM (r = 0.306, *p* = 0.019) (Table 6).

**Table 6:** Correlation coefficient between the set of serum markers and the clinical data

	MR		Procalcitonin		Presepsin		MCP-1	
	r	P value	r	P value	r	P value	r	P value
**PRISM** ^**(P)**^	***0.950***	< 0.001*	***0.536***	< 0.001*	*0.051*	*0.702*	***0.306***	0.019*
**LOS** ^**(P)**^	*−0.015*	*0.913*	*− 0.026*	*0.846*	*0.122*	*0.363*	*−0.206*	*0.121*
**Mortality** ^**(S)**^	***0.861***	< 0.001*	***0.474***	< 0.001*	*0.125*	*0.350*	*0.178*	*0.181*
**Ventilatory Support** ^**(S)**^	***0.795***	< 0.001*	***0.422***	0.001*	*0.132*	*0.322*	*0.086*	*0.519*
**Low GCS** ^**(S)**^	***0.812***	< 0.001*	***0.473***	< 0.001*	*0.038*	*0.777*	*0.106*	*0.427*
**Inotropic drug use** ^**(S)**^	***0.732***	< 0.001*	***0.305***	0.020*	*−0.026*	*0.845*	*0.105*	*0.434*

(P) Pearson’s correlation

(S) Spearman’s correlation

*: Significant level at *P* value < 0.05

*PRISM* Pediatric Risk of Mortality score, *LOS* Length of Stay, *GCS* Glasgow Coma Scale

## Discussion

Physicians at critical care centers have the data that guide them to diagnose infections in newly admitted patients and differentiate it from non-infectious presentations. Sometimes, full physical examination and medical history are sufficient for the diagnosis of sepsis [3]. However, in comatosed patients or in some cases of non-infectious SIRS conditions (e.g., trauma, hemorrhages, burns, pancreatitis, hypothermia, and surgery) the diagnosis of sepsis becomes difficult. In these conditions, several suggested new tests can assess the possibilities of various illnesses to be differentiated from sepsis.

The mortality rate in our study was 43.1% in sepsis group and 37.5% in SIRs group. It was less than that reported by El Hamshary et al. who reported that mortality was 72% in a study carried in Egypt [18]. Yet, another Egyptian study done by Rady, reported a similar mortality rate of 33.1% [19]. Variable rates of mortality in these studies can be explained by different criteria of admission, infection control measures, nursing staff experiences and equipment facilities. Generally, the PICUs in developed countries had lower mortality than those in developing countries [20].

Till now, no single biomarker has the absolute diagnostic capability to differentiate sepsis from SIRS, to monitor response or predict prognosis [21]. In this study, we assessed the diagnostic and discriminative capabilities of four biomarkers of sepsis: sMR, PCT, Presepsin, and MCP-1 to distinguish sepsis from severe SIRS in critically ill newly admitted patients with signs of severe acute inflammation on admission.

To the best of our knowledge, this is the first study that evaluate the diagnostic value of these four biomarkers of sepsis. The major finding of this study, performed in critically ill newly admitted patients with suspected infection, was the good discriminative power of sMR, Presepsin, and MCP-1 to differentiate between sepsis and SIRS and the confirmation of the formerly reported high diagnostic accuracy of PCT. The data obtained are consistent with several studies, as will be mentioned for each biomarker later.

All the four markers increased significantly in sepsis group compared to both the SIRS and control groups. To the authors’ knowledge, MCP-1 has been scarcely investigated in sepsis, studies done by Bossink et al. and Wang et al. who observed an elevation in the serum level of MCP-1 in adult sepsis [22, 23].

A longitudinal, prospective, and observational study on 15 patients with sepsis was done by Sans et al. the study was at baseline and on days 1, 2, 5, 7 and 10 of their stay in the ICU. They observed that the MCP-1 concentration significantly decreased with the resolution of sepsis, and this decrease was especially important during the first 5 days of hospitalisation [24].

Our study addresses for the first time, the possible role of MCP-1 as a possible predictor of sepsis in children with Sensitivity 100% and Specificity 62.5%.

Our findings shed the light on the value of sMR as new serum biomarker for pediatric sepsis.

Hansen et al. concluded that levels of sMR elevated in several diseases, including sepsis and liver disease, thus sMR shows promise as a new biomarker [25].

Vlieger et al. showed that serum sMR concentrations were higher in critically ill patients with infections than those with non-infectious inflammation [12].

PCT has been proven by many studies to be superior to CRP in the diagnosis of sepsis and intensity of infection. Our study showed that PCT a good predictor of sepsis with the AUC of 0.966 (0.9-0.993) with high sensitivity and fair specificity at a cut-off point of 0.62 ng/ml. Many researchers have studied the diagnostic and prognostic exactness of PCT measurement [26].

Recent studies have shown that PCT is not as a good predictor of sepsis as newly examined biomarkers in adult and pediatric sepsis and septic shock [7, 27].

Presepsin was recognized as a novel biomarker for sepsis in many studies, most of them were performed on neonates with sepsis [28, 29]. Shozushima et al. found that the concentration of Presepsin was significantly lower in the SIRS group than in the sepsis group [30].

Yoon et al. stated that Presepsin has higher both sensitivity and diagnostic accuracy, but lower specificity than PCT or CRP in detecting sepsis in children (AUC of presepsin was 0.925 with sensitivity 0.94 and specificity 0.71) [31].

Bellos et al. concluded that the use of Presepsin in the early neonatal period in high-risk populations as its diagnostic accuracy seems to be high in detecting neonatal sepsis (AUC of presepsin was 0.9751 with sensitivity 0.91 and specificity 0.91) [32].

Other studies have found that the plasma levels of Presepsin in infected patients were higher than that in non-infected patients [33].

In our study, Presepsin was higher in children with sepsis. This coincides with Contenti et al. who studied the concentration of Presepsin in emergency patients on admission [14].

This study has many limitations and strengths. First, strengths include blind investigation of cases by clinicians without awareness of the level of the biomarkers. Also, contrary to other studies [34, 35]. Only patients with a strong probability of sepsis were included, this has covered the patients that are likely to be faced with the use of these diagnostic tests.

While limitations include that our patients’ number was not very high. Another limitation is that our findings may not be applicable to patients with mild illness or localized infections that do not require ICU admission as in these cases, the levels of the biomarkers may be below the submitted cutoff values.

## Conclusion

Although our study included a relatively small number of patients, it suggests a potential role for sMR, Presepsin, and MCP-1 as biomarkers in differentiating between sepsis and SIRS in critically ill children, so more studies should be done on a larger number of children. Furthermore, our results confirmed the former reports that PCT is one of the most useful sepsis biomarkers in critically ill children. These findings could direct clinicians in their practical decision-making and complex management of severely ill children who need much interference in short time.

### Impact on society

Biomarkers as sMR, Presepsin, and MCP-1 can be used to differentiate between sepsis and SIRS in critically ill children. That may help in taking urgent treatment decisions.

## Data Availability

All datasets used and/or analyzed during the current study are available from the corresponding author on reasonable request.

## Abbreviations

ALT: Alanine aminotransferase
AUC: Area Under the Curve
CI: Confidence Interval
CNS: Central Nervous System
CONS: Coagulase Negative Staphylococcus
CRP: C Reactive Protein
DIC: Disseminated Intravascular Coagulation
EDTA: Ethylenediaminetetraacetic Acid
GCS: Glasgow Coma Scale
ICU: Intensive Care Unit
IQR: Interquartile Range
LPS-LBP: lipopolysaccharide- Lipopolysaccharide Binding Protein
MCP-1: Monocyte Chemoattractant Protein-1
MODS: Multiple Organ Dysfunction Syndrome
NPV: Negative Predictive Value
PCT: Procalcitonin
PICU: Pediatric Intensive Care Unit
PPV: Positive Predictive Value
PRISM: Pediatric Risk of Mortality score
qSOFA: Quick Sequential Organ Failure Assessment
ROC: Receiver Operating Characteristic Curve
S-MR: Soluble Mannose Receptor
SD: Standard Deviation
SIRS: Systemic inflammatory response syndrome
SPSS: Statistical Package for Social Sciences
